# Potential of *Schisandra chinensis* (Turcz.) Baill. in Human Health and Nutrition: A Review of Current Knowledge and Therapeutic Perspectives

**DOI:** 10.3390/nu11020333

**Published:** 2019-02-04

**Authors:** Adriana Nowak, Małgorzata Zakłos-Szyda, Janusz Błasiak, Agnieszka Nowak, Zhuo Zhang, Bolin Zhang

**Affiliations:** 1 Institute of Fermentation Technology and Microbiology, Lodz University of Technology, Wolczanska 171/173, 90-924 Lodz, Poland; agnieszka.nowak@p.lodz.pl; 2 Institute of Technical Biochemistry, Lodz University of Technology, Stefanowskiego 4/10, 90-924 Lodz, Poland; malgorzata.zaklos-szyda@p.lodz.pl; 3 Department of Molecular Genetics, Faculty of Biology and Environmental Protection, University of Lodz, Pomorska 141/143, 90-236 Lodz, Poland; janusz.blasiak@biol.uni.lodz.pl; 4 Beijing Forestry University, College of Biological Science and Biotechnology, No. 35 Qinghua East Road Haidian District, Beijing 100083, China; zhuoz329@163.com (Z.Z.); zhangbolin888@163.com (B.Z.)

**Keywords:** *Schisandra chinensis*, anti-cancer effect, anti-aging potential, anti-obesity activity, anti-diabetic action

## Abstract

*Schisandra chinensis* (Turcz.) Baill. (SCE) is a plant with high potential for beneficial health effects, confirmed by molecular studies. Its constituents exert anti-cancer effects through the induction of cell cycle arrest and apoptosis, as well as inhibition of invasion and metastasis in cancer cell lines and experimental animals. SCE displays antimicrobial effects against several pathogenic strains. It has anti-diabetic potential, supported by hypoglycemic activity. A diet rich in SCE improves pancreatic functions, stimulates insulin secretion, and reduces complications in diabetic animals. SCE prevents lipid accumulation and differentiation of preadipocytes, indicating its anti-obesity potential. SCE exerts a protective effect against skin photoaging, osteoarthritis, sarcopenia, senescence, and mitochondrial dysfunction, and improves physical endurance and cognitive/behavioural functions, which can be linked with its general anti-aging potency. In food technology, SCE is applied as a preservative, and as an additive to increase the flavour, taste, and nutritional value of food. In summary, SCE displays a variety of beneficial health effects, with no side effects. Further research is needed to determine the molecular mechanisms of SCE action. First, the constituents responsible for its beneficial effects should be isolated and identified, and recommended as preventative nutritional additives, or considered as therapeutics.

## 1. Introduction

For many years, natural plants have been used in nutrition, food production, and medicine. Many of them contain an array of compounds with antimicrobial, antioxidative, anti-proliferative, and anti-cancer activity [1]. Natural plant compounds have the potential to induce pro-health effects, resulting in an extension of life expectancy and improvement of its quality. Plant extracts and plant-derived compounds can improve the properties of functional food with their well-documented pro-health effects [2]. 

*Schisandra chinensis* (Turcz.) Baill. (SCE) is a plant whose fruits have a long-standing use in traditional Chinese medicine. They have been used in the treatment of diseases of the gastrointestinal (GI) tract, respiratory failure, cardiovascular diseases, body fatigue and weakness, excessive sweating, and insomnia [3]. They were also reported to reduce hunger, delay aging, increase vitality, and improve mental health [4]. They demonstrate neuro and hepato-protective, anti-inflammatory, antioxidative, detoxification, immunostimulant, antiviral, and anti-cancer activities, as well as cardiovascular and skin-protective properties [5,6,7,8]. 

Reviews of the pro-health effects of SCE focus primarily on their influence on the central nervous, sympathetic, cardiovascular, endocrine, and respiratory systems, as well as its adaptogenic, hepatoprotective, immunostimulant, antioxidant, ergogenic, and anti-stress activities [3,4,9,10,11]. This review provides updated information on the phytochemical composition of SCE and the mechanisms underlying its beneficial activity in humans, including its anti-cancer, antimicrobial, anti-diabetic, anti-obesity, and anti-aging actions. 

## 2. Biologically Active Compounds in SCE 

*S. chinensis* contains many bioactive compounds, including lignans, triterpenes, phenolic acids, flavonoids, essential oils, and polysaccharides. Lignans are mainly responsible for the pro-health properties of SCE. These compounds are predominant in SCE fruits, but can also be found in the leaves, shoots, and seeds. They were extracted from the biomass of in vitro cultures [12,13,14]. The most widely represented groups of SCE lignans are dibenzocyclooctadiene lignans, which, due to structural similarity to and occurrence in plants of the *Schisandra* genus, are often referred to as “schisandra lignans”. Within dibenzocycloactadiene lignans, which occur in the largest amounts in the fruits of *Schisandra chinensis,* are schisandrin (syn. schisandrol A, wuweizisu A), schisandrin B (syn. gomisin N, wuwezisu B, γ-schisandrin), schisantherin A (syn. gomisin C, schisandrer A), schisantherin B (syn. gomisin B, schisandrer B), schisanhenol (syn. gomisin K3), deoxyschisandrin (syn. schisandrin A), and gomisin A (syn. schisandrol B) [4]. A WHO (World Health Organization) monograph [15] stated that about 30 *Schisandra* lignans were identified, but to ensure the pro-health activity of fruits, their content should not be lower than 0.4%. At present, many more lignans in SCE have been detected. For example, schineolignins A–C, belonging to the butane-type lignans dibenzyl group, were described by Xue et al. [16]; and schilignan F (tetrahydrofuran lignan) was isolated by Yang et al. [17], from rattan stems of SCE. 

The chemical composition and resulting biological activity of plant extracts depends on humidity, light, soil type, latitude, season, maturity, harvest time, geographical location, temperature, and other factors [18]. Additionally, the content of individual lignans in SCE fruits depends on the location of the crop, the degree of fruit maturity, and harvest season [19,20,21]. Zhang et al., (2009) studied ten fruit samples from different provinces of China [19]. In six of them, schisandrin was predominant (2.199–5.332 mg/g), while, in the other four, schisantherin A (2.263–6.36 mg/g) dominated. Thirty fruit samples, examined by Liu et al., showed the highest content of schisandrin (3.51–11.08 mg/g) [20]. This compound constituted 31%–33% of the *Schisandra* lignans in fruits originating from Korea, and 36%–46% of those from China. In eight out of ten fruit samples, tested by Wang et al., the relationship in the concentration of SCE lignans was schisandrin > gomisin A > schisandrin B [21]. 

Another important group of biologically active compounds isolated from SCE is the triterpenoids. They constitute a broad and structurally diverse group of chemical compounds. SCE contains lanostane and cycloartane-type triterpenoids and nortiterpenoids, which, in the scientific literature, are often termed as "*Schisandra* nortriterpenoids" or schinortriterpenoids [22]. An example of a compound belonging to the lanostane-type triterpenoids is kadsuric acid, described by Yang et al. [23]. Examples of cycloartane-type triterpenoids are schisanlactone D and wuweizilactone acid [23,24]. Schinorterpenoids are isolated from different parts of the plant—fruits (schindilactone A, wuweizidilactone I), leaves (schindilactones IK, wuweizidilactones JP, schisanartanin N), rattan stems (schindilactone LM, wuweizidilactone S), and roots (schinchinelactone D) [16,23,25,26,27].

Flavonoids and phenolic acids, which are polyphenols, display antioxidant properties. They are secondary plant metabolites, which occur in every part of the plant (i.e., fruits, flowers, seeds, leaves, roots, or even lignified parts). Among phenolic acids, Mocan et al., found chlorogenic acid in the fruits of SCE, while in the leaves two other derivatives of hydroxycinnamic acid (*p*-cumaric and ferulic) were found [28]. Significantly more compounds from this group were detected by Szopa et al. [29]. These authors found chlorogenic acid and five hydroxybenzoic acid derivatives: gallic, *p*-hydroxybenzoic, protocatechuic, syringic, and vanilic acids, in the leaves and fruits. Flavonoids present in SCE fruits are isoquercitin, quercetin, and its derivatives—quercetin 3-galactoside (hyperoside) and quercetin 3-rutinoside (rutin). SCE leaves also contain quercetin 3-ramnoside (quercitrin) myricetin and kaempferol [13,29]. Fruits of SCE also comprise the cyanidin derivatives: Cyanidin-xylosylrutinoside, cyanidin-glucosylrutinoside, cyanidin-xylosylglucoside, and cyanidin-rutinoside, belonging to the anthocyanins [30,31,32]. 

SCE fruits also contain essential oils. The content of individual groups of compounds can be put in the following order: Sesquiterpene hydrocarbons > oxygenated sesquiterpenes > oxygenated monoterpenes > monoterpene hydrocarbons. The main aromatic compounds are ylangene (11.93%–37.71% of the volatile fraction), α-himachalene (18.03%–20.7%), and β-himachalene (6.29%–10.46%) [33,34].

Finally, polysaccharides isolated from SCE fruits have been intensively studied. SCE is the source of homogeneous polysaccharides composed mainly of glucose, galactose, mannose, and rhamnose in various molar proportions. Their mass ranged from 18 to 127 kDa [35,36,37,38]. Polysaccharides also occur in combination with uronic acid and proteins [39,40,41]. 

SCE fruits contain substantial amounts of minerals. Sowndhararajan et al., showed that 100 g of dried fruits contains Fe, Mn, Cu, K, and Mg in amounts that cover 96%, 320%, 48%, 54%, and 33% of the Recommended Daily Intake (RDI) of these ingredients, respectively [34]. According to the European Union legal regulations, a food product can be treated as a source of a particular substance if it contains more than 15% of the RDI of that substance in 100 g of the product [42].

## 3. Health Promoting Effects of SCE Constituents 

A rich chemical composition and the presence of diverse biologically active compounds make SCE a plant with strong potential to induce pro-health effects, and its use in disease treatment is subsequently the subject of intense research.

### 3.1. Antimicrobial Activity 

So far, SCE berry extract has demonstrated antibacterial effects against several Gram-positive and Gram-negative bacteria. Oils from SCE seeds showed a good antibacterial activity against *Escherichia coli, Bacillus cereus*, *Enterobacter aerogenes, Serratia marcescens*, and *Micrococcus luteus,* as tested by the disc diffusion method [43]. Teng and Lee [43] investigated the efficacy of various extraction methods, but simultaneous distillation extracted higher amounts of terpenes, β-pinene, borneol, and α-pinene, as well as limonene, than other procedures, including Soxhlet and microwave-assisted extraction. These compounds might display a strong antibacterial activity due to the penetration through the outer membrane of bacterial cells and its severe damage. Six dibenzocyclooctadiene lignans presented antibacterial activity against pathogenic *Chlamydia pneumoniae* and *Chlamydia trachomatis* upon their infection in human epithelial cells [44]. The presence and substitution pattern of methylenedioxy, methoxy, and hydroxyl groups of the lignans had a profound impact on the antichlamydial activity [44]. Bai et al., investigated the activity of SCE fruit ethanolic and water extracts against typical food-borne pathogens and food-spoiling organisms [45]. Both extracts displayed strong antibacterial activity towards *Staphylococcus aureus*, *Listeria monocytogenes*, *Bacillus subtilis*, *B. cereus*, *Salmonella enterica* subsp. *enterica* serovar Typhimurium, *Pseudomonas aeruginosa*, *Enterobacter aerogenes* and *E. coli* [45]. It was suggested that the main constituents responsible for such activity were organic acids (such as citric and malic acids), as evaluated by ion chromatography. Mocan et al. evaluated the minimal inhibitory concentration (MIC) of *S. chinensis* fruit and leaf extracts for the Gram-positive *S. aureus*, *B. subtilis*, and *L. monocytogenes*, and the Gram-negative bacteria *E. coli* and *S.* Typhimurium, which ranged from 10 µg/mL to >100 µg/mL [13]. These results indicated that Gram-positive bacteria were more sensitive to SCE extracts than Gram-negative bacteria. The same was observed by Choi et al., for methanol fractions of SCE against several Gram-negative (*S.* Typhimurium, *E. coli, Cronobacter sakazakii*) and Gram-positive (*B. cereus, L. monocytogenes, S. aureus)* strains [46]. This difference may result from the difference in cell wall morphology of these microorganisms [46]. Reports on the stimulation of microbial growth by compounds from SCE are less abundant. In a conference report, *S. chinensis* rhizome extract was reported to promote growth of *Lactobacillus delbrueckii* ssp. *bulgaricus*, while inhibiting activity of *Bacillus licheniformis*, *B. subtilis*, and the pathogenic *E. coli* [47]. The mechanism of inhibition includes changing the permeability of the outer membrane of bacteria, leading to their destruction [47]. 

### 3.2. Anti-Cancer Effect

**Figure 1:**
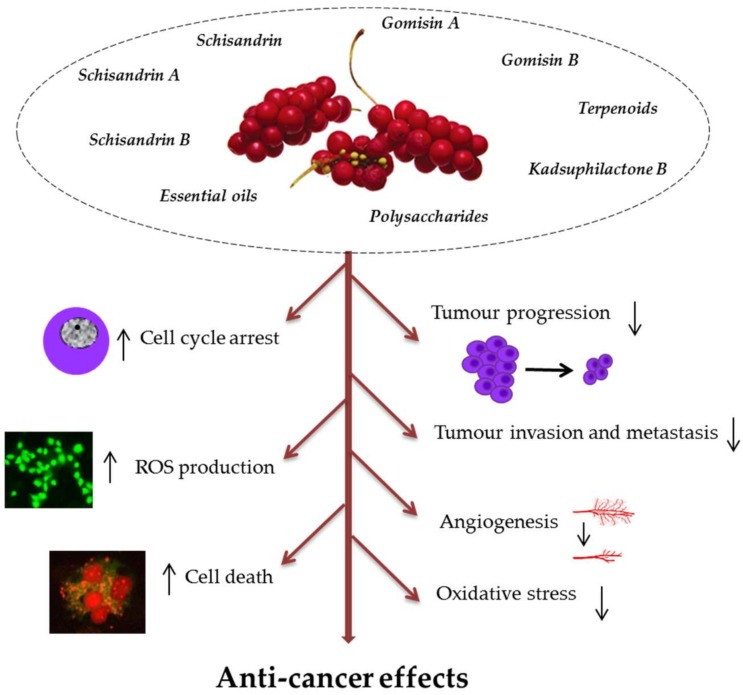
Mechanisms of anti-cancer activity of bioactive phytochemicals in *Schisandra chinensis* (SCE). They may inhibit tumour progression through cell cycle arrest at G0/G1 and G2/M, suppression of proliferation, invasion, metastasis, and angiogenesis. SCE antioxidative action includes induction of the antioxidant enzymes and direct scavenging of reactive oxygen species (ROS) to prevent cancer induction and progression. Their pro-oxidant effects lead to increased ROS production in cancer cells and cell death (apoptotic and autophagic).

Currently, therapeutics of botanical origin are of high interest in the treatment of cancer and many other diseases. The anti-cancer activity of polyphenols from plant extracts in cancer cell lines includes several mechanisms: Inhibition of tumour proliferation, induction of cell death (apoptotic, autophagic), inhibition of tumour migration and invasion, cell cycle arrest, pro-oxidant activity by stimulation of ROS (reactive oxygen species) production in cancer cell lines, as well as reducing oxidative stress in normal cells and inhibition of carcinogen activity [48]. The main mechanisms of anti-cancer action of SCE phytochemicals are presented in Figure 1. 

Cytotoxic (anti-proliferative) activity of SCE main constituents, such as gomisin N, was demonstrated against many cancer cell lines [5,49,50,51,52]. When overdosed, SCE is toxic—the minimal toxic dose when given orally to mice is 3.6 g/kg [3]. Normal cell lines, in reaction to plant-derived chemical compounds (such as phytochemicals and essential oils), can behave differently to cancerous cells [53]. There are limited data on cytotoxicity and genotoxicity of *Schisandra* extracts or its constituents in normal cell lines, compared to their cancer counterparts. Kee et al., demonstrated the anti-cancer activity of gomisin A, through inhibition of proliferation of several colorectal cancer cell lines, whereas that compound did not change the proliferation of normal colon cells [54]. Some studies show an ability of main constituents of SCE to induce cell cycle arrest and apoptosis in cancer cells by ROS-mediated/mitochondria-dependent pathways [49,51,55,56,57]. Schisandrin B has been shown to protect against oxidative damage in liver, heart, and brain tissues in rodents [58,59]. Cell migration and invasion are critically involved in cancer metastasis, the main cause of death in cancer patients [60]. In in vivo research, schisandrin B attenuated cancer invasion and metastasis in BALB/c mice (an albino, laboratory-bred strain of the house mouse useful for research into both cancer and immunology) [61]. In in vitro studies, schisandrin B inhibited the invasion and migration of the human alveolar basal epithelial adenocarcinoma cell line (A549) by down-regulating the expressions of hypoxia inducible factor (HIF-1), vascular endothelial growth factor (VEGF), and matrix metalloproteinases (MMP-2 and MMP-9) [51]. Then, gomisin A reduced invasion and migration of colorectal cancer cell lines, as well as the metastasis in BALB/c mouse lung [54]. The research relevant to mechanisms of cytotoxicity and apoptosis of SCE in cancer cells is advanced, while data on normal cells are limited and in vivo data are insufficient. Recent studies on the mechanisms of anti-cancer action of SCE are listed in Table 1. 

### 3.3. Anti-Obesity and Anti-Diabetic Action

Due to its antioxidant, hepatoprotective, and anti-cancer activities, SCE fruit has been used as a traditional medicine for treatment of various cardiovascular or GI ailments in South-Eastern Asia and Russia [71]. Recently, the interest in its application as a preventive agent against diet-related chronic diseases, such as type 2 diabetes (T2D), obesity, or non-alcoholic fatty acid disease (NAFLD), has increased. 

#### 3.3.1. SCE as A Carbohydrate Metabolism Modulator 

**Figure 2:**
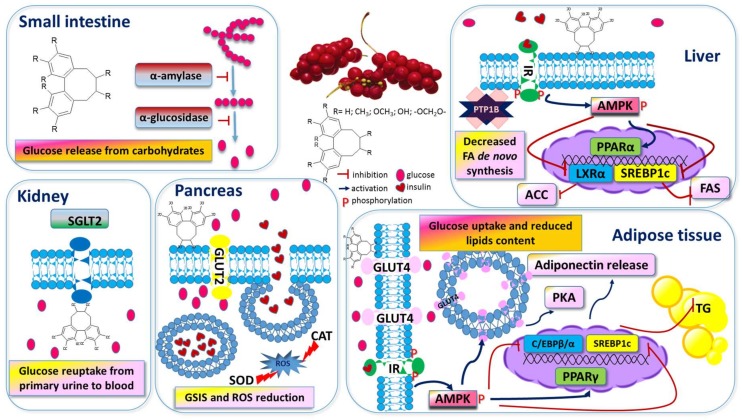
Proposed molecular mechanisms of anti-diabetic and anti-obesity actions of *Schisandra chinensis* in different organs and adipose tissue. See main text for more details. ACC—acetyl-CoA carboxylase; C/EBPβ/α—CCAAT/enhancer-binding protein alpha; CAT—catalase; FAS—fatty acid synthase; GLUT2, 4—glucose transporter 2, 4; GSIS—glucose stimulated insulin secretion; HSL—hormone sensitive lipase; IR—insulin receptor; LXR α—liver X receptor α; PKA—protein kinase A; PTP1B—protein tyrosine phosphatase 1B; ROS—reactive oxygen species; SOD—superoxide dismutase; SREBP-1c—sterol regulatory element binding protein 1c; and TG—triglyceride.

One of the main features of T2D is a chronic elevation of blood glucose level, known as postprandial hyperglycaemia. Predominantly after meal intake, glucose is released from carbohydrates in the digestive tract. That reaction is catalysed primarily by α-amylase, which hydrolyses starch to maltose and maltotriose, as well as α-glucosidase, which catalyses glucose release from disaccharides and oligosaccharides [72]. Thus, the inhibition of these activities is used as the first therapeutic target to control blood glucose level. Jo et al., checked in vitro the inhibitory potential of two SCE water polyphenolic extracts, fruit pulp, or skin and seeds and demonstrated that the former was a potent inhibitor of porcine pancreatic α-amylase and rat intestinal α-glucosidase (Figure 2) [73]. These hypoglycaemic properties were confirmed further, during in vivo studies, when a SCE preparation decreased blood glucose level in rats after sucrose solution oral administration, with an efficiency higher than observed for acarbose—a known α-glucosidase inhibitor.

Blood glucose level is strongly influenced by its transport and absorption in the small intestine and reabsorption in the kidneys, as well as uptake in other peripheral tissues. In these processes, two types of membrane-integrated transporters, known as glucose transporters (GLUTs) or sodium glucose cotransporters (SGLTs), are involved [74]. SGLT1 is involved in intestinal glucose absorption, whereas SGLT2 and SGLT1 take part in the renal reabsorption of filtered glucose from the primary urine. In diabetic patients, due to the elevated levels of SGLT2 expression, there is increased renal glucose reabsorption leading to hyperglycaemia. Therefore, inhibitors of SGLT2 transporters are considered as potential diabetes treatments. Data presented by Qu et al., showed that the fraction obtained from SCE fruit ethanol extract enriched with lignans—deoxyschisandrin, schisandrin B, and schisandrin—selectively inhibited glucose reuptake by SGLT2, which finally eliminated glucose excess via urine, leading to a decrease in blood glucose level [74,75]. Among known GLUTs responsible for the uptake of different monosaccharides, the GLUT4 isoform is the principal protein involved in glucose transport into insulin-sensitive tissues [76]. In T2D patients, the cellular expression of GLUT4 is reduced, indicating a lower capability to process glucose in vivo [77]. GLUT4 is mainly expressed in muscle cells and adipocytes, but it has been recently found in other types of cells [75]. It has been shown that a low molecular weight polysaccharide fraction extracted from SCE up-regulates the expression of GLUT4 in fibroblasts of hepatic origin (Buffalo Rat Liver) and improves glucose uptake [75]. However, cellular glucose uptake by the GLUT4 transporter requires translocation from intracellular space to the plasma membrane and is regulated by 5’-adenosine monophosphate-activated protein kinase (AMPK) [78]. AMPK is one of the most important regulators of lipid and glucose metabolism, and is regarded as a potential target in the prevention and treatment of diabetes (Figure 2). AMPK is activated via its phosphorylation under metabolic stress, when ATP consumption and the AMP:ATP ratio increase. Jin et al. have confirmed that the hypoglycaemic activity of SCE polysaccharides was associated with the increase of pAMPK protein level, as well as the mRNA of *IRS-1, GLUT-4*, *AMPKα*, and *PPAR-γ* levels [75]. On the other hand, dephosphorylation of the insulin receptor (IR) and insulin receptor substrate IRS-1 by another enzyme, protein tyrosine phosphatase 1B (PTP1B), interrupts the insulin signalling pathway. Thus, PTP1B inhibitors increase insulin sensitivity and glucose tolerance, and a petroleum ether extract of SCE was recognised as a PTP1B phosphatase inhibitor [79].

Pancreatic beta cells secreting insulin play a critical role in glucose homeostasis. Due to an intrinsic low level and the minimal activity of the antioxidant enzymes catalase (CAT), glutathione peroxidase, and superoxide dismutase (SOD), these cells are very sensitive to oxidative stress induced by elevated levels of glucose and free fatty acids [72] (Figure 2). Thus, the high antioxidant activity and radical-scavenging capacity of SCE extracts play an important role in the protection of beta cells against their dysfunction or death [73]. It was observed, in diabetic rats, that SCE oil supplementation improved pancreatic β-cells function by upregulation of SOD and CAT, along with the expression of the anti-apoptotic *Bcl-2* gene [80]. Moreover, due to the hyperglycaemia and oxidative stress, the impairment of insulin release from granules by beta cells and loss of pancreatic mass were observed [81]. Insulin acts as an agonist for insulin receptors (IR), and in turn, activates further glucose uptake by other peripheral tissues, including adipocytes, via GLUT4. A water extract of SCE showed insulinotropic action to improve glucose-stimulated insulin secretion by mouse Min6 cells [81]. However, further analysis on diabetic rats, subjected to overnight fasting, suggested that a SCE preparation improved glucose homeostasis by the promotion of insulin sensitivity, but not secretion capacity [81]. In accordance with that conclusion, recent studies demonstrated SCE protective activity against the production of advanced glycation end-products (AGEs), which are harmful to endothelial cells [82]. AGEs are produced through non-enzymatic reactions during hyperglycaemia and promote ROS production, which attenuates the expression and activity of endothelial nitric oxide synthase (eNOS), leading to a reduced NO level, endothelium dysfunction, and finally to the formation of atheromatous plaques. It was shown that human umbilical vein endothelial (HUVEC) cell incubation with SCE elevated the expression and activity of eNOS, via downregulation of RhoA/Rho kinase activity; therefore, SCE was able to reduce hyperglycaemia-induced microvascular complications [82].

#### 3.3.2. SCE as A Lipid Metabolism Modulator 

Obesity is characterised by the accumulation of excess fat in adipose tissues, and its development occurs along with the differentiation of preadipocytes into adipocytes. This process is known as adipogenesis, and can be observed in vitro by the increased ability of adipocytes to accumulate triacylglycerol in cytosolic lipid droplets. Schisandrin B reduced lipid content, as well as increased acid oxidation and lipolysis, with the activation of protein kinase A (PKA) and hormone sensitive lipase (HSL) in 3T3-L1 adipocytes [83] (Figure 2). The same work showed a similar influence of schisandrin B on subcutaneous mouse adipocytes in an ex vivo model. In an in vivo experiment, schisandrin B reduced subcutaneous adipocytes size, subcutaneous adipose tissue mass, and the body weight in mice. 

The major and essential adipogenesis regulator is peroxisome proliferator-activated receptor gamma (PPAR-γ) (Figure 2). Insulin sensitizers are its agonists, able to increase triacylglycerol storage in adipose tissues by increasing insulin-stimulated glucose uptake. Sterol regulatory element binding protein 1c (SREBP-1c) is another important transcriptional factor involved in lipid metabolism. Activation of PPAR-γ and SREBP-1c regulates the expression of other genes which encode proteins responsible for fat cell development, considered as a positive effect that will stimulate β-oxidation, insulin sensitivity, adiponectin secretion, glucose uptake, and catabolism. It was demonstrated that SCE fruit extract, enriched with schisandrin, gomisin A, and angeloylgomisin H, acted as a PPAR-γ agonist and improved insulin resistance in the liver [84]. However, further studies showed that SCE extract and its lignans prevented lipid accumulation and impaired the differentiation of 3T3-L1 preadipocytes into adipocytes, via downregulation of the key adipocyte differentiation regulatory genes *PPARγ* and *C/EBPβ/α* [85,86]. The same study confirmed that SCE anti-obesity properties observed in vitro were comparable to results obtained in high fat diet (HFD)-induced obese rats. 

The liver plays a major role in maintaining normal blood glucose levels by regulating *de novo* glucose production and glycogen breakdown. Hepatic insulin resistance results in the elevation of hepatic glucose production and triglyceride (TG) accumulation (by impairing insulin-mediated inhibition of gluconeogenesis and regulating insulin-mediated TG metabolism, respectively), which contributes to hyperglycaemia and dyslipidaemia. Therefore, the control of hepatic insulin resistance is an attractive therapeutic target for treating T2D and hepatic steatosis. Studies, performed on hamsters treated with a high fat diet, showed that SCE ethanol extract decreased food and energy intakes, lowered body and fat weights (as well as the serum TG and LDL levels), insulin resistance, inflammation, and liver steatosis [71]. The mitigation of liver lipid accumulation was due to the increase in *PPARα* mRNA level and decrease of the transcription of lipogenic *SREBP-1c* that promotes lipid synthesis in the liver and is involved in triacylglycerol synthesis through fatty acid synthase (FAS) and acetyl-CoA carboxylase (ACC) (Figure 2). At the protein level, the extract considerably increased the AMPK phosphorylation (pAMPK), which activated cellular lipid oxidation allowing ATP generation with the simultaneous exclusion of energy-consuming processes, such as TG and protein synthesis [71]. Furthermore, a SCE lignin extract inhibited FAS enzymatic activity [87].

Chronic excessive lipid accumulation in the liver leads to the development of non-alcoholic fatty liver disease (NAFLD). It was shown that a SCE water extract lowered hepatic triglyceride, total cholesterol, glucose content, and ameliorated ALT (alanine aminotransferase) release by a liver injured by fenofibrate (a PPARα agonist) in normal and hypercholesteraemic mice [88]. Recent studies demonstrated that a SCE ethanol extract attenuated palmitic acid and oleic acid-induced lipid accumulation in human hepatoma HepG2 cells [89]. Zhu et al., showed that SCE decreased the expression of ER stress markers and inflammatory genes encoding Il-6, TNF-α, and MCP-1 proteins, whereas Chung et al. revealed that SCE berry ethanol extract ameliorated lipid accumulation by the inhibition of histone acetyltransferase (HAT) through the inhibition of total lysine acetylation [88,90]. The authors observed decreased levels of *Pparγ, Srebp-1c*, and *Fas* gene expression in mice supplemented with SCE [90]. The hepatoprotective activity of SCE was also revealed by a polysaccharide fraction, which downregulated mRNA and protein expression of SREBP-1c, FAS, ACC, and liver X receptor α (LXRα) in mice with induced NAFLD. In addition, gomisin N, derived from SCE, activated AMPK phosphorylation in steatotic HepG2 cells, which consequently led to LXR restrain, deactivation of ACC (acetyl-CoA carboxylase) via phosphorylation, and the prevention of SREBP-1c translocation [91]. In the presented pathway, active LXRα, after binding to the retinoid X receptor (RXR), stimulates the expression of SREBP-1c and its target lipogenesis genes by binding to LXR response elements in their promoters [92]. Therefore, reduction of triglyceride accumulation in adipocytes and hepatocytes may result from the multifaceted biological activity of SCE.

### 3.4. Aging-Related Effects of SCE

Organismal aging is determined by many factors besides the date of birth, but cellular senescence can be an important element of aging and age-related diseases [93,94]. Several physiological and pathological conditions are subjects of aging research, including senescence, direct aging, photoaging, oxidative, mitochondrial, and inflammatory aging, among others. To study aging in experimental practice, either cellular replicative senescence is investigated or various animal models of aging are used. D-galactose-induced aging is one (if not the most) commonly applied model of aging [95]. However, it should be considered as a model of accelerated, rather than physiological, aging. It is out of the scope of this review to consider conceptions and models of aging. Instead, some effects of SCE in biological systems, which can be related to aging, will be considered.

Schisandrin B and its analogue, schisandrin C, were shown to protect human and rat foreskin fibroblasts against oxidative damage induced by artificial solar light [96,97]. These substances were proposed to be used in the prevention of skin photoaging. They exerted a protective effect by the stimulation of the production of reduced glutathione, decreased expression of matrix metalloproteinase 1, and an elastase-type protease. However, these compounds also produced ROS during their metabolism, mediated by the cytochrome P-450, and this reaction likely provoked potentiated antioxidant response by the glutathione system. Similar results were obtained for schisandrin B in the human keratinocyte-derived cell line HaCaT [98]. Schisandrin B reduced the cell death, DNA damage, and oxidation of proteins in these cells challenged by oxidative stress; and increased the expression of key enzymes of the antioxidant defence and stimulated the Nrf2 (nuclear factor erythroid 2-related factor 2) and MAPKs (mitogen activated protein kinases) pathways. Similar effects were observed for deoxyschisandrin and schisandrin B in HaCaT keratinocytes exposed to UVB. Altogether, these effects were concluded to be important in the prevention of skin aging underlined by oxidative stress.

Osteoarthritis (OA) is a joint disease, affecting the middle-aged to elderly [99]. An ethanol extract of SCE was shown to exert a protective effect against cartilage degradation in a monosodium iodoacetate (MIA)-induced OA rat model [100]. This protection was underlined by a reduced production of inflammatory cytokines and tumor necrosis factor-alpha (TNFα), an inhibited expression of inducible nitric oxide synthase and cyclooxygenase-2, and increased levels of matrix metalloproteinase-13, cartilage oligomeric matrix protein, and a C-telopeptide of type II collagen. 

Sarcopenia, a progressive loss of muscle strength and mass with aging, is commonly considered as an important indicator of normal aging and occurs in some diseases associated with accelerated aging [101]. SCE was shown to increase mass of skeletal muscle in mice and rats treated by dexamethasone or that underwent sciatic neurectomy [102,103,104,105]. To explore the mechanisms beyond these effects, Kim et al. showed that SCE ameliorated muscle atrophy by increased protein synthesis resulting from downregulation of the mTOR/p-4E-BP1 (4E-binding protein1)/p-P70S6K (70 kDa ribosomal protein S6 kinase) pathway in human myoblasts [106]. However, SCE can also promote protein degradation through the FOXO1/MuRF1 pathway, but its net action resulted in muscle hypertrophy. A former work of Kang adds some information on this mechanism, pointing at heme oxygenase-1 (HO-1) and Nrf2, which can be targeted by SCE in C2C12 myoblasts [107]. As aging compromises muscle mass, amelioration of these effects by SCE can be considered as a manifestation of its anti-aging potential. In their recent work, Kim et al. showed that SCE upregulated genes whose products are important in protein synthesis and muscle growth in old mice after chronic forced exercises (swimming) [108]. Additionally, SCE downregulated genes important for protein degradation. SCE also reduced the levels of ROS and lipid peroxidation, as well as upregulating some antioxidant enzymes and inhibited certain apoptotic markers. Therefore, SCE can be considered as an element to assist an exercise-based, healthy life style. Similar conclusions can be drawn from the experiments showing that omija fruit extract as a diet supplement improved the running endurance of rats [102]. That work also showed an upregulation of peroxisome proliferator-activated receptor gamma coactivator 1-alpha (PGC-1α), and some other proteins in the skeletal muscle of trained animals.

Gomisin A, another bioactive compound isolated from SCE, was shown to suppress stress-induced premature senescence and the production of proinflammatory molecules in human fibroblasts [109]. This effect was attributed to the promotion of mitochondrial biogenesis and autophagy by gomisin A in these cells, as well as its antioxidant activity. However, some aspects of that work need clarifying, including the determination of the reasons and consequences of the observed effects.

Diet supplementation with schisandrin B was shown to ameliorate age-related impairment of mitochondrial antioxidant functions in various tissues of C57BL/6J mice [110]. This suggests that schisandrin B can increase the survival of aging individuals by improvement of mitochondrial functions. However, despite convincing results on the mutual relationship between aging and antioxidant mitochondrial function in rats, this relationship cannot be directly translated to humans [111].

Rats with accelerated aging, induced by D-galactose, fed with a diet rich in SCE lignans showed the expression of 15 biomarkers of antiaging mechanisms [112]. The markers were involved in energy, amino acid, lipid, and phospholipid metabolism, and almost all returned to the control levels after termination of SCE lignan supplementation. Moreover, a SCE lignan-rich diet resulted in mRNA overexpression of the p19, p53, and p21 proteins in the brain of aging animals. Therefore, these metabolic changes in SCE lignan-fed rats can be underlined by the modulation of the expression of these proteins and become an element of antiaging prevention and therapy.

Aging is not only associated with a decline in biochemical functions, but also in behavioural/cognitive performs [113]. Yan et al., showed that a D-galactose-induced rat, with diet supplemented with ethanol extracts of SCE partitioned with petroleum ether, ameliorated cognitive deficits assayed by the Morris water maze and Step-down type passive avoidance test, as compared to animals with non-supplemented diet [114]. These behavioural changes were associated with a decreased activity of antioxidant enzymes induced by D-galactose and a normal level of oxidative stress markers, including glutathione, malondialdehyde, and nitric oxide in the serum and various structures of the brain of treated animals [114].

**Figure 3:**
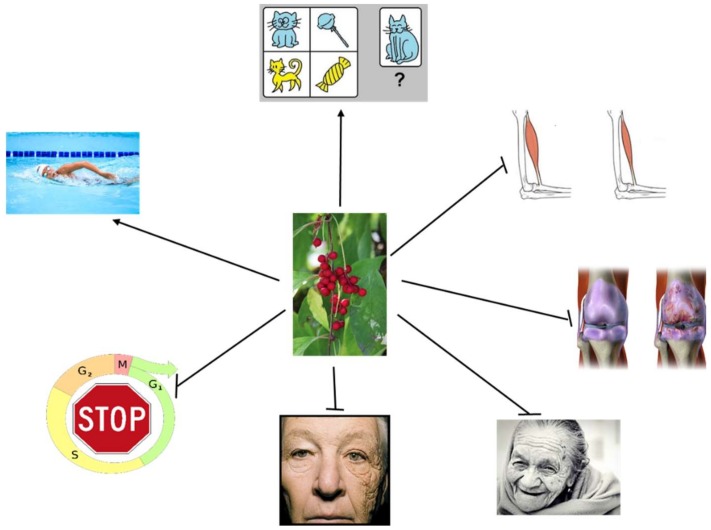
*Schisandra chinensis* and its derivatives can modulate aging-related phenomena in humans, experimental animals and cell cultures. They can suppress skin photoaging, ameliorate sarcopenia and osteoarthritis, increase physical endurance, inhibit stress-induced premature senescence, improve cognitive and behavioural functions, and modulate other effects that can also be associated with a delay of normal aging.

In summary, SCE, its extracts, and derivatives can display beneficial effects against pathological aspects of aging in various systems used to investigate aging mechanisms, including cell cultures and animals (Figure 3). How these effects can be related to human aging and age-related diseases remains to be determined, but they justify further research into the anti-aging properties of SCE. 

## 4. Conclusions and Future Perspectives

*S. chinensis* is known as a Chinese-originated important phytochemical source, with many beneficial biological activities. About 8% of the world adult population suffer from type 2 diabetes, almost 13% are obese, and 39% are overweight [115,116]. Cancer is the second leading cause of death globally, responsible for an estimated 9.6 million deaths in 2018 [117]. Some of the reasons for this are low fruit and vegetable intake, bacterial and viral infections, and obesity/overweight. Additionally, cancer, in many cases, can be considered as an aging disease, though the mechanisms underpinning that relationship remain unclear. 

SCE extracts, as well as its individual constituents—including lignans, triterpenes, phenolic acids, flavonoids, essential oils, and polysaccharides—display several anti-cancer mechanisms in vitro, such as suppressing cell proliferation, cell cycle arrest, triggering apoptotic death, and inhibition of the invasion and migration of cancer cells. Nonetheless, in vivo research seems to be insufficient [50,51,52,54,56,57,62,63,64,65,66,67,68,69,70]. Both SCE and its bioactive phytochemicals demonstrate antimicrobial activity, depending on extract concentration and microbial strain. A few works have proven that SCE exhibits anti-viral activity [118,119,120]. 

SCE phytochemicals are involved in carbohydrate- and lipid-metabolism regulation. The presented review updates knowledge on the molecular mechanisms in which SCE lignans, polyphenols, and polysaccharides are implicated (e.g., in the regulation of transcriptional factors and enzyme activities, or the selective inhibition of glucose transporters). The anti-diabetic and anti-obesity abilities of SCE can be recommended in the prevention or control of metabolic disorders, in the form of a programmed dietary supplement or functional food component. 

SCE can also exert several effects that can be interpreted as the expression of its anti-aging properties. However, the influence of this compound on normal life-span has not been studied thus far. Moreover, in many experiments, the anti-aging effects of SCE can be concluded based on aging markers of various specificity. Therefore, indicators that are more aging-specific could be subjected to further research. Furthermore, most of the anti-aging effects attributed to SCE are associated with its antioxidant properties, but the process of aging—both normal and accelerated—should not be limited to the increased production of ROS.

As the human microbiome is a potential mediator of the influence of dietary intake on metabolic status, little attention has been given to the role of SCE in the modulation of the human gut microbiota, and thus, the subsequent pro-health effects. In one randomised study, an increased abundance of the genus *Ruminococcus* in obese women associated with lower fruit intake and higher metabolic syndrome risk was demonstrated [121]. Also, there is no research on the effects of co-administration of probiotics with SCE. As the composition of the intestinal microbiota considerably changes with aging and the related disease outcomes, it is important to determine the role of SCE in these processes. As metabolism of plant extracts by human gut microbiota has become a subject of extensive investigation, the future perspective is in the isolation, identification, and estimation of the biological activity of the metabolites of SCE released after digestion in the human GI tract, and also after supplementation with probiotic bacteria. 

A major problem with research on natural substances and their products is in the standardisation, as it is difficult to compare results from different laboratories. This is especially important when effects associated with natural plants are studied. They contain many substances, which may induce diverse biological effects, depending not only on the phenotype and genotype of the target object, but also on the location of their growth, as soil and air composition may contribute to the composition of plant tissue. Moreover, it should be considered that the effect induced by a single product isolated from a plant may have little to do with the effects evoked by the total extract of that plant. Therefore, two basic methods can be considered—isolation and purification of a single product from a plant, and the use of its extract. The former is relatively simple to standardise: The degree of purity should be established, along with a maximal allowed concentration of specific impurities. Standardisation of the extraction procedure is much more complex, and it should include determination of the part of a plant, its age, development, soil composition, climate, season, chemicals added, and so on. Therefore, the only way to follow these requirements is to cultivate a plant in optimal, determined conditions, but then, such conditions must be regulated, and the plant is no longer “natural”. 

The role of SCE in cellular reaction to oxidative stress appears to be especially important in its biomedical applications. Oxidative stress is implicated in the pathogenesis of many diseases, and can be associated with many environmental/life style factors [122]. It was shown that SCE stimulates antioxidant enzymes, mainly SOD and glutathione peroxidase [123]. However, antioxidant enzymes are not the only components of cellular antioxidant defence. Low-molecular weight antioxidants and DNA repair are other elements of that system, and little is known about SCE’s influence on them. Moreover, it is not completely known whether some chemical components of SCE have the ability to directly scavenge ROS through their chemical inactivation. Choi showed that schisandrin A prevented DNA damage, apoptosis, and mitochondrial dysfunction induced by hydrogen peroxide, and these effects could be associated with lowering the H_2_O_2_ levels in C2C12 cells [124]. However, it was not determined which mechanism was directly responsible for that lowering. Moreover, it was shown, in that work, that schisandrin A lowered the extent of the phosphorylated h2AX histone, which is a marker of the repair of DNA double-strand breaks. Therefore, a multifaceted action of schisandrin A against genotoxic effects could be considered, and it is important in future research to keep that in mind and not to limit the report to only final or intermediate consequences of the antioxidant effects of SCE. These three main elements of cellular antioxidant defence: Antioxidant enzymes, low-molecular weight antioxidants, and DNA repair, should be considered and investigated in the context of the antioxidant action of SCE, starting from its ability to directly inactivate a stress-inductor without the involvement of any cellular structure. 

An apparent lack of side effects or cumulative action is often mentioned as a major advantage of SCE use. It was stated that SCE extracts in ethanol are non-toxic for experimental animals, even at very high doses [3]. However, most in vitro research on the beneficial effects of SCE in a disease is performed on normal or cancer cell lines, and the target disease is modelled by introducing elements of its pathogenesis. This is not a reliable model to investigate the human response to toxic doses of SCE in various pathologies. Moreover, interaction of SCE with other drugs should also be investigated to avoid unwanted effects.

The beneficial effects of SCE on physical endurance, assessed as the extension of the time taken to continuously swim or run, should be more closely associated with its effect on energy production [102]. It seems that PGC-1α, a major regulator of mitochondrial biogenesis, is a candidate to interact with SCE to promote more effective physical efforts, but further studies on that subject are needed, as PGC-1a fulfils many important functions, including antioxidant defence [125,126]. Therefore, the beneficial effect of SCE on physical activity would be associated with directed energy production and distribution, as well as with the fighting of fatigue-induced oxidative stress through interaction with PGC-1a, but this hypothesis needs further verification.

Cognitive and behavioural functions are also reported to be improved by SCE. These properties are, in general, associated with antioxidant effects and an improvement of energy metabolism in the brain [127]. Therefore, model cells and animals for the study of neurodegenerative diseases can be suitable for research on some aspects of such beneficial aspects of SCE. However, there are some problems with establishing the causative relationship between the reason and consequences of oxidative stress and annotating the influence of SCE. These functions, in improving cognitive and behavioural functions, are closely associated with the anti-aging functions of SCE, as many degenerative diseases and defects are age-related.

## Figures and Tables

**Table 1:** Recent studies on the anti-cancer action of *Schisandra chinensis* (SCE) constituents.

SCE constituent/extract	In vitro effects	Reference
Schisandrin B	Inhibition of triple negative breast cancer growth by inducing cell cycle arrest/apoptosis.	[52]
Schisandrin B	Cell cycle arrest at G0/G1, apoptosis, inhibition of invasion, and migration of A549 cells.	[51]
Sesquiterpenes from fruits (ethanol extract)	Cytotoxicity in Caco2 cells.	[50]
Dibenzocyclooctadiene lignans (+)-deoxyschisandrin (1) and (−)-gomisin N (2), from *S. chinensis* fruits	Inhibition of adenocarcinoma cells (ovarian 2008 and colon LoVo) growth by apoptosis correlated with G2/M arrest and tubulin polymerisation.	[55]
Polysaccharides	Apoptosis of renal cell carcinoma Caki-1 by blocking secretion of endothelial growth factor.	[62]
Polysaccharide-0-1	Inhibition of human hepatocellular liver carcinoma (HepG2) cells growth via G2/M arrest/apoptosis.	[56]
Polysaccharides	Mitochondria-mediated apoptosis in human renal carcinoma cell line (Caki-1) through the inactivation of the extracellular-signal-regulated kinase (ERK) pathway.	[63]
Semen essential oil	Apoptosis of human leukaemia U937 cells by ROS- and caspase-dependent mitochondrial dysfunction and nuclear translocation of mitochondrial pro-apoptotic proteins.	[64]
Gomisin G	Suppression of proliferation and cell cycle arrest in G1 in triple-negative breast cancer cells.	[65]
Gomisin A	Decreased viability of various colorectal cancer cell lines; G0/G1 arrest and apoptosis of CT26 and HT29 cells by regulating cyclin D1/cyclin-dependent kinase 4 (CDK4) expression and apoptotic proteins; reduced invasion and migration of cancer cells.	[54]
Deoxyschisandrin from fruits (ethanol extract)	G0/G1 arrest in human ovarian cancer cells (A2780, SKOV3, and OVCAR3); reduced protumoural phenotype of tumour-associated macrophages (TAMs).	[57]
Deoxyschisandrin Schisandrin B	Protection against UVB-induced DNA damage and apoptosis in HaCaT cells via ROS scavenging.	[66]
Fruit water extract	Inhibited proliferation, depolarised mitochondrial membrane and apoptosis by ROS generation and activation of caspase-9 and -3 in AGS human gastric cancer cells.	[67]
Terpenoids from the n-hexane fraction of *S. chinensis* extract	Cytotoxicity in human leukemic HL-60 cells.	[68]
Nineteen compounds isolated from hexane and ethyl acetate soluble fractions from fruits (ethanol extract)	Cytotoxicity in human ovarian (A2780) and endometrial cancer (Ishikawa) cells.	[69]
Kadsuphilactone B	Apoptosis via the Bcl-2 (B-cell lymphoma 2) and MAPK (mitogen activated protein kinase) signalling pathways.	
**In vivo effects**		
Schisandrin B	Inhibition of migration and colony formation of tumour cells, prevention of growth of TNBC cells in mice via suppression of signal transducer and activator of transcription-3 (STAT3) phosphorylation and nuclear translocation.	[52]
Polysaccharides	Antitumor and antiangiogenic activity associated with the up-regulation of Bax and p53, downregulation of Bcl-2, as well as the reduction of CD31 and CD34 in tumours in BALB/c nude mice.	[62]
Gomisin A	Inhibition of metastasis of colorectal cancer in a lung metastasis mouse model.	[54]
Seed extract	Apoptosis in human osteosarcoma cells (1547).	[70]
